# Advances in Twin-Screw Granulation Processing

**DOI:** 10.3390/pharmaceutics13050624

**Published:** 2021-04-27

**Authors:** Uttom Nandi, Vivek Trivedi, Steven A. Ross, Dennis Douroumis

**Affiliations:** 1 Faculty of Engineering and Science, School of Science, University of Greenwich, Chatham Maritime, Chatham, Kent ME4 4TB, UK; u.nandi@kent.ac.uk; 2 CIPER Centre for Innovation and Process Engineering Research, Kent ME4 4TB, UK; steven@cubi-tech.co.uk; 3 Medway School of Pharmacy, Medway Campus, University of Kent, Central Avenue, Chatham Maritime, Chatham, Kent ME4 4TB, UK; V.Trivedi@kent.ac.uk; 4 Cubi-Tech Extrusion: 3, Sextant Park, Neptune Cl, Rochester ME2 4LU, UK

**Keywords:** twin-screw granulation, granulation mechanisms, PAT tools, QbD, continuous processing

## Abstract

Twin-screw granulation (TSG) is a pharmaceutical process that has gained increased interest from the pharmaceutical industry for its potential for the development of oral dosage forms. The technology has evolved rapidly due to the flexibility of the equipment design, the selection of the process variables and the wide range of processed materials. Most importantly, TSG offers the benefits of both batch and continuous manufacturing for pharmaceutical products, accompanied by excellent process control, high product quality which can be achieved through the implementation of Quality by Design (QbD) approaches and the integration of Process Analytical Tools (PAT). Here, we present basic concepts of the various twin-screw granulation techniques and present in detail their advantages and disadvantages. In addition, we discuss the detail of the instrumentation used for TSG and how the critical processing paraments (CPP) affect the critical quality attributes (CQA) of the produced granules. Finally, we present recent advances in TSG continuous manufacturing including the paradigms of modelling of continuous granulation process, QbD approaches coupled with PAT monitoring for granule optimization and process understanding.

## 1. Introduction

Granulation is a well-known powder processing technique, used in the pharmaceutical industry for the manufacturing of solid dosage forms [1]. This term is used to describe the processing of powders for particle enhancement with the aim to improve a range of properties such as flowability, compressibility, tabletability and the homogeneous distribution of active ingredients, etc. [2,3]. The transformation of powders to agglomerated particles is usually performed prior to tableting to ensure minimal aggregation and a uniform flow of processing material from the feeding hooper, and good compressibility in the tablet dies [2]. Typically, granulation is a particle size enlargement process where particles are processed to form larger multi-unit entities with uniform distribution ranges, from smaller sized granules with a distribution in between 0.2–0.4 mm, to larger sized granules with (around 1–4 mm) typically prepared for capsule manufacturing [4,5]. Depending on the final particle size, granules are further processed with other excipients and used as an intermediate before compression to form tablets, or for filling hardened gelatine capsules. Furthermore, granulation technology is used to reduce the generation of toxic dust during powder handling [5]. A good quality granule exhibits less a non-friable behaviour and produces less fines during processing. Pharmaceutical powders often have uneven particle size distribution leading to segregation during storage which can be minimized and improve content uniformity via granulation. The bulk powders present several practical implications such as higher packaging, storage and transportation requirements which can be tackled through the manufacturing of granules with higher densities.

In general, pharmaceutical manufacturing can be divided into two distinct stages: primary manufacturing (i.e., upstream operations), the production of the active ingredient from the starting reagents, and secondary manufacturing (i.e., downstream operations), which involves the conversion of the active drugs into products suitable for administration such as granules, capsules or tablets [6]. The secondary manufacturing involves the integration of multiple processing units, necessitating an comprehensive understanding of processing parameters to optimise the formulation [7,8,9].

The manufacturing of granules can be conducted via three different approaches: dry, wet and melt granulation. The dry granulation involves the aggregation of primary powders in the absence of any liquids, under high pressure, to facilitate the bonding of the particles by direct contact followed by milling to attain the desired size [1]. This method is suitable for processing moisture sensitive active pharmaceutical ingredients (APIs), due to the absence of a liquid component. Another benefit to this method is its cost-effectiveness due to the fewer processing steps, little or no material waste and low dust exposure. A common approach for dry granulation is roller compaction where powder is fed into two counter rotating rolls producing flat shaped ribbons of compacted material. The use a dry binder, such as cellulose, starch or povidone, in the powder blend is essential to achieve a stronger ribbon [10]. Subsequently, the ribbons can be milled to obtain the desired granule size. In many cases, the use of roller compaction/dry granulation has been described as a “continuous production line” [11]. The technique has been frequently used over the past two decades and has been shown to improve drug dosage weight control, content uniformity and powder flow, while also being easy to scale up and facilitating continuous manufacturing [12]. However, this technique does result in greater generation of fines and requires high forces for compaction, making the technology unsuitable for the manufacture of certain drugs e.g. processing of highly potent drugs increase the chances of environmental contamination while poorly compressible drug substances may lead to sticking on the rollers [13,14].

A wide range of powder processing methods are employed in the pharmaceutical industry to produce granules with particular characteristics in terms of their physical and pharmaceutical properties (e.g., bioavailability) [15]. According to several studies, wet granulation provides better control of drug uniformity, improves flowability and increases bulk density and porosity [16,17,18]. Also, volatile and non-toxic solvent (e.g., ethanol, isopropanol) can be used for the processing of powder blends which are an alternative for moisture sensitive APIs as they tend to dry quickly. The use of aqueous solution is a common approach to create strong bonds between the particles that lock them together. However, when the water dries the powders might break and thus it is a prerequisite to form a dense mass. Following, the production of granules, further processing is carried out by sieving, milling or mixing with additional components to manufacture a finished dosage form. Wet granulation technologies usually involve the use of high shear mixers and fluidize bed that produce dense and uniform granules.

**Figure 1:**
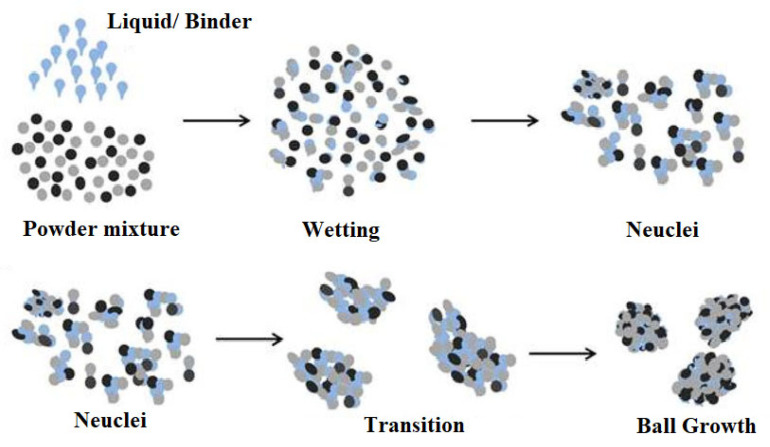
Diagram of granule formation (Reproduced with permission from [19], Elsevier, 2019).

Theoretically, granule mechanism requires the adhesion of particles for the formation of agglomerates and comprises of three key steps. The particle adhesion takes in various steps, as shown in Figure 1. This involves wetting and nucleation, transition and ball growth [5]. Initially, adhesion or cohesion forces take place through the formation of an immobile thin layer which results in the decrease of the interparticulate distance while increasing the contact area though Van der Waals forces. This is the pendular state where water is distributed as mobile thin layer around the particles holding them together through lens-shaped rings. The transition state is entered by the further addition of granulating liquid leading to the formation of stronger liquid bridges (funicular state) by filling up the interparticulate void space. This is usually the end of the wet granulation process but further liquid saturation (80%) and air displacement lead to the capillary state where large spherical granules (ball growth) are built up which are not suitable for pharmaceutical purposes.

Melt granulation is similar to the dry granulation process, however, the binder solution is replaced with a meltable binder. Fluidised bed melt and melt extrusion or even high shear granulations are processing technologies that can be effectively used where the binder is melted/soften near or above its melting point or glass transition temperature in order to obtain agglomerated granules [20,21,22,23]. Usually, the melting point of binders varies between 30–100 °C and they either melt or become tacky followed by solidification upon cooling.

In this review, we provide a comprehensive discussion on twin-screw granulation processing including granulator (extruder) details, the effect of processing parameters on the granule production followed by the formation mechanisms of the different granulation approaches. Recent advantages on continuous twin-screw granulation are also presented along with case studies of QbD, and Process Analytical Tools (PAT) implementation by various research groups.

## 2. Instrumentation of a Twin-Screw Granulator (Extruder) (TSG)

Extrusion granulation equipment is almost identical to that used for typical Hot Melt Extrusion (HME) and consists of the extruder, auxiliary equipment, downstream processing equipment and some other monitoring tools for the performance and evaluation of products [24]. The extruder typically is composed of a feeding hopper, single or twin screw, barrels, die and driving unit for screw. The auxiliary equipment includes cooling and heating device connected to the barrels, the conveyer belt to cool down the extrudates and the solvent delivery pump. The monitoring screen displays screw speed, temperature scales, torque monitor and pressure scales.

Overall extrusion process in divided into four sections [25]:

- Feeding of the materials
- Conveying of materials (mixing and particle size reduction)
- Out flow from the extruder (no die)
- Downstream processing

**Figure 2:**
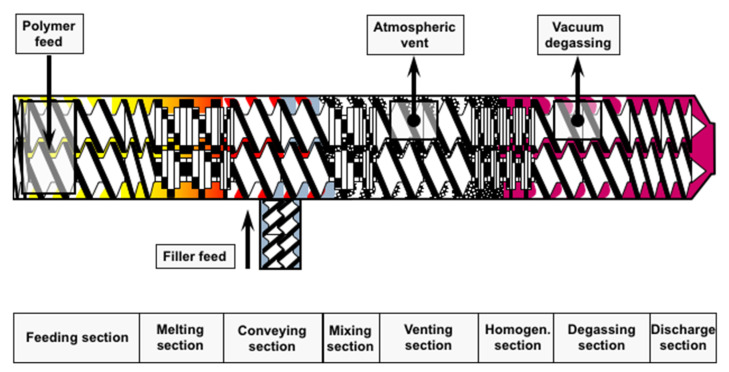
Schematic diagram of an extruder (image was kindly offered by Coperion GmbH).

During processing, the temperature is controlled by electrical heating bands and monitored by thermocouples displayed on monitoring screen. Usually, the extruder comprises of single or twin rotating screws placed inside the barrel. The barrel is comprised of sections which are bolted and clamped together and described as zones in the extruder. A die plate is attached to the end of barrel, which determines the shape of final extruded product (Figure 2).

Currently, the most common extruder types are: (a) the single screw and (b) the Twin Screw extruders [26]. The single screw extruder consists of only one screw placed in the extruder barrel while more advanced twin screw extrusion systems consist of a pair of screws that rotate in the same direction (co-rotating) or opposite direction (counter rotating). Usually, the extruder screw is characterised by length/ diameter (L/D) ration of screw which typically ranges from 20:1 to 40:1.

The heat required (melt granulation) for the melting or fusing of the material is provided by the combination of electric or liquid clams on the barrels and friction of the materials produced by shear force between rotating screw and barrel wall [27,28].

The extrusion granulation process is fully controlled by applying the optimal temperature, screw speed and feed rate. The technique has a potential to control and process material at specific requirements such as high shear extrusion or addition of solvent at solvent evaporation stage during processing. Screw configuration allows the extruder to perform mixing and particle size re-arrangement which performs a vital role for the dispersion of API into carrier matrices.

**Figure 3:**
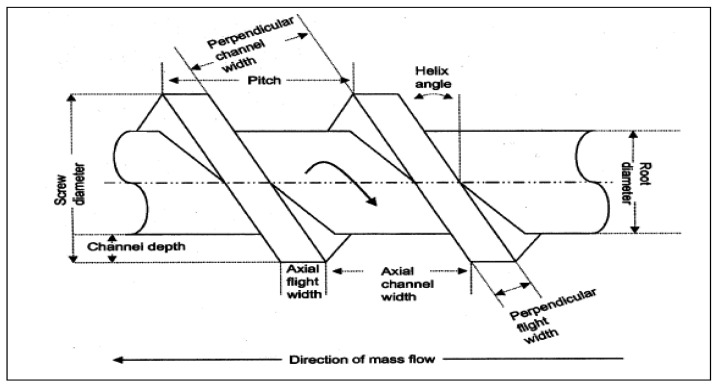
Geometry of an extruder screw (Reproduced with permission from [25], Elsevier, 2002).

The material is fed from the hopper directly into the deeper flights or greater flights pitch. The geometry of flights (shown in Figure 3) allows the feeding materials to fall easily into to rotating screw for conveying alongside the barrel. The helix angle and pitch of the screw controls the constant rotation speed of screws. The materials are fed as a solid into the transition zone where it is mixed, compressed, melted and plasticized. The compression process of materials in the barrel is regulated by either decreasing thread pitch but maintaining constant flight depth or vice versa [29]. This process creates pressure as the material moves through the barrel towards the discharge zone. This discharge zone is similar to these conveying elements, except the channel depth is far greater to allow for the build-up of more material, to allow for quick discharge. The material moves in a helical path as a result of transverse flow, pressure flow, drag flow and leakage while the movement of material in barrel is affected by two reverse mechanisms (a) screw diameter space and (b) width of the barrel. The material reaches the metering zones with uniform thickness and flowability which drag out the granulated material as a uniform delivery of granules. The downstream processing is equipped with conveyer which permits extrudates to cool down to room temperate by applied air pressure.

**Figure 4:**
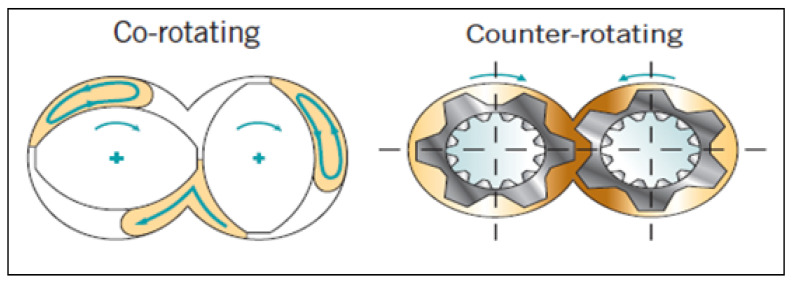
Twin screw co-rotating and counter-rotating screw (reproduced with permission from [31], IOPscience under Creative Commons Attribution 3.0 licence).

In twin screw extruders, screws can be rotating either in the same (co-rotating) or opposite (counter-rotating) direction as shown in Figure 4. Two screws placed into the barrel side by side and it is designed to control operating parameters such as the filling level of material, the screw speed, the feed time and the residence time [30]. In a counter rotating extruder, twin screw rotates in opposite direction in barrel. This type of extruder is usually applied for high shear materials as material squeezed through the gaps between screws. A counter rotating screw provides an excellent dispersion of blended particles, however, it also leads to increased air entrapment, high pressure and low output as a result of the lower maximum screw speed. This type of extruder does not produce a pushing effect of materials into the barrel due to opposite direction of screw rotation, so material throughput is purely dictated by the feed rate, as powder moves through the counter rotating screws due to being pushed through by the powder behind it.

On the other hand, a co-rotating screw rotates in the same direction and it has the advantage of a self-wiping and intermeshing design [25]. This type of extruder is mainly used in the industry which has competency to operate at high screw speed and provide excellent high output with intensive mixing and conveying of materials during process. Co-rotating is sub-classified as non-intermeshing and intermeshing.

Intermeshing twin screw extruders are very popular as they provide self-wiping to minimize the non-motion of materials, prevent overheating and display superior mixing compared to single screw extruders. Intermeshing extruders operate on the principle of first in/first out where material does not rotate along with screw to barrel [32]. Non-intermeshing extruders are used to extrude a highly viscous material which is not liable to cause high torque during processing [33]. This type of counterrotating screw configuration is more popular in plastic or polymer processing industry, as heating during the operation substantially reduces torque generation.

## 3. TSG Processing Parameters

### 3.1. Temperature

The heating for each zone of the barrel is uniformly and accurately controlled, therefore, the process can be run using a single temperature value or a temperature profile, depending on the specific requirements of the process. Temperature has an effect on the processability of the formulation, as viscosity is temperature dependent, and also on the quality of the final product. The bed temperature for lubricated powders varies from 5–15 °C and depends on the flow rate, screw speed, L/S ratio and the amount of polymer in the binding solution [34,35]. The temperature variations within the barrel can easily reach 70 °C while the presence of long mixing zones increases the temperature of the formed granules. The temperature rise in the barrel can be also influenced by the different wetting behaviour of the processed powders [36]. For example, the high absorption capacity of microcrystalline cellulose resulted in non-uniform wetting of the solids and prevented sufficient process lubrication leading to increased frictional heating.

### 3.2. Screw Design

Screw configuration is designed based on the type, number and sequence of phases a given processing material requires during manufacture. At the entrance of the extruder, conveying screw elements of high pitch sizes are placed to ensure proper powder feeding since bulk density of the inlet materials is much smaller than extrudate density. A standard configuration presents conveying elements up to two third of the screw length, followed by high a shear mixing zone. Venting during the extrusion process is sometimes needed to remove entrapped air or residue moisture from the final product. Venting requires a drop in pressure in that part of the extruder to prevent the exiting of the material through the opening. This is achieved through the positioning of high pitch conveying elements in the barrel area meeting the venting zones.

**Figure 5:**
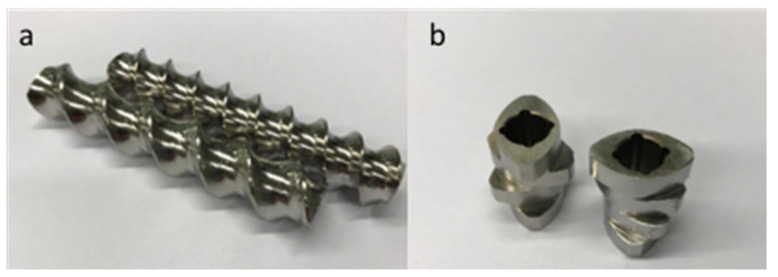
Conveying elements (**a**) and mixing elements (**b**).

The elements on the screw shaft are interchangeable so a customised screw configuration can be assembled to match the specific requirements of each process. Elements differ in design to suit the various steps of processing, such as transporting, mixing, melting and shaping. Conveying elements have self-wiping geometry needed for transportation of the material along the screw, whereas the free volume of the screw and speed is modulated by pitch size (Figure 5). Mixing (kneading) elements are used for mixing, melting and homogenisation while they comprised of disc, which are staggered under certain angle. The angle determines the conveying and mixing properties; conveying properties decrease whereas mixing properties increase with increasing offset angle. Additionally, longer discs impart more shear, while shorter improve dispersive mixing. Therefore, kneading elements can be classified as:

- forwarding (convey polymer to the die)
- neutral (used for either distributive or dispersive mixing)
- reversing (convey polymer flow back toward the feed)

Screw configuration has a critical impact on the product properties where insufficient mixing results in non-homogenous blends or leads to incomplete product conversion or reaction. On the contrary, if the mixing zone is too long and imparting too much shear, residence time and temperature can increase, resulting in API degradation.

The use of conveying elements with different flight pitches can have a significant effect on the extruded granules. By increasing the flight pitch of the screws, the granule output is increased while the fines are reduced due the larger volume which is available for the wetted mass [37,38]. The use of higher flight pitch facilitates the formation of porous granules while the increased number of conveying elements results in higher granule strength. The presence of conveying elements right after a kneading zone plays a key role as it minimizes the formation of large clumps, resulting into their breakage.

**Figure 6:**
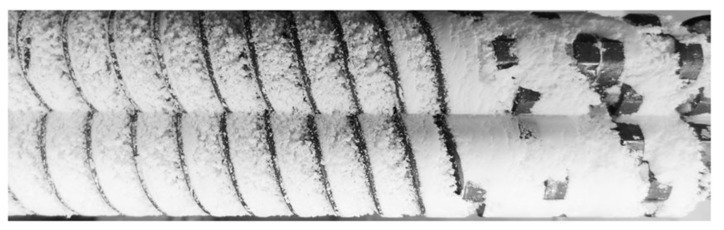
Photo showing a wet twin-screw granulation (TSG) wet of microcrystalline cellulose/lactose monohydrate mixture with a kneading block (right most side of the image). The direction of flow is to the right.

Furthermore, the kneading elements play a key role due to their capability to produce stronger compressive granules. The Figure 6 show a TSG process illustrating the granule development and compaction. When using high offset angles of kneading elements granules appear larger and denser while longer mixing zones produced less friable and breakable particles with narrow and controllable size.

To the contrary the usage of multiple kneading zones has a negligible effect on the granule particle size and distribution. Kneading elements control the morphology of extruded granules and usually produce elongated shapes compared to spherical shaped granules obtained when only conveying screws are implemented [39,40].

### 3.3. Feeding Rate and Screw Speed

The feeding rate is one of the most important process variables and it has been found to influence the particle size and granule density/strength. High feeding rates are related to increased compressive forces which correspond to larger, dense granules. In the absence of kneading zones, high feed rates increase the formation of fines which are friable and easily break while the liquid distribution in the powder blend is non-uniform. Screw speed is related to the shear rate in a TSG, but it has a low impact on the particle size of the granules and the residence time. By increasing the screw speed only a minor size reduction has been observed and only when high adhesive polymer are used in the granulation blend [41].

### 3.4. Mixing in a TSG

The powder mixing in a TSG is directly related to the screw configuration and the resulting shear rate as a function of the process settings. This is classified as radial or axial based on the direction of spread. During the TSG process radial mixing is a prerequisite for a homogeneous granule distribution at constant powder feeding while axial mixing can also help to avoid inhomogeneities. The selection of feeding rate, screw speed and screw configuration determine the degree of mixing. The axial mixing is directly affected by the Residence Time Distribution (RTD), screw speed and geometry. When using high screw speeds, the axial mixing increases as estimated by the rise of the normalized variance (σ2θ) and the lowering of the Peclet number (Pe). However, there are contradictory studies suggesting that axial mixing is not affected by the processing parameters [42].

The most favourable feature of a TSG process is the capacity to process high powder throughput in a short residence time (0–20 s). Despite the possible gains of TSG over batch manufacturing this is not always straightforward because there is a limited time frame to achieve homogeneous granule distribution, granule formation and breakage to obtain the final product. Hence, in order to achieve a high yield, the process should be designed carefully with longer residence time than the mixing time across the extruder barrel. The process is more complicated when mixing powders with the granulating liquid which requires homogeneous liquid distribution within the powder. Sayin et al. observed that the site of liquid addition and the periodicity of the peristaltic pump has a strong impact on the moisture content and distribution [43]. The use of more kneading zones can help to improve the distribution of granulation liquid, but further improvements require the addition of screw elements with modified geometries that can induce distributive mixing.

The addition of granulating liquid results in the formation of liquid bridges between the powder particles and subsequently aggregation takes place to produce larger granules. The evolution of particle size and the primary shaping mechanism in the TSG is limited due to the capacity of the extruder. Extensive studies have shown that granule size increases not only after the kneading zones but also upstream (before) suggesting there are not only two granule formation mechanism such as the dispersive and distributive mixing. In the third mechanism the built-up material before the kneading zone (flow restriction) is forced- mixed with the inbound powder due to the throughput force. As a result, the increase in the throughput (feeding) lead to the increase of granule size assuming there is enough granulating liquid to from large agglomerates.

Kumar et al. investigated the interrelations of the residence time and mixing settings over the quality of extruded granules [44]. The study demonstrated that the increase of kneading elements had no influence on the yield fraction despite the improvement of powder mixing and residence time. The same was observed by increasing the L/S ratio which resulted only in the formation of larger granules.

## 4. Twin Screw Granulation

**Figure 7:**
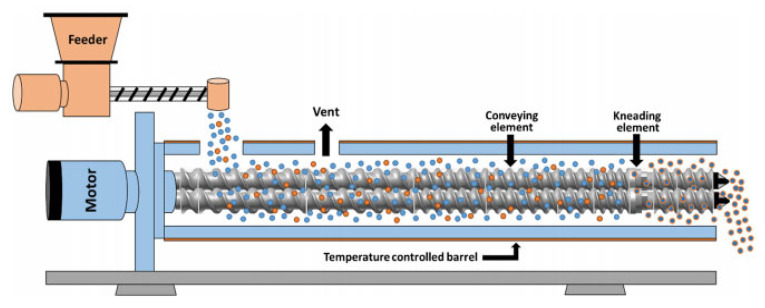
Schematic representation of a twin screw granulator (Reproduced with permission from [46], Elsevier, 2020).

TSG is an advanced processing technology that has been extensively used for granules production over the last couple of decades [45]. Several studies have been reported relating to TSG optimization by investigating the effect of the formulation composition and operational variables such as screw configuration, pitch and length of conveying element, thickness and angle of kneading element, or influence of kneading blocks. A schematic diagram of a TSG line is illustrated in Figure 7.

The first study for the development of pharmaceutical granules at a laboratory scale using a single-screw extruder was reported by Goodhart et al. in 1973 [47]. The investigation was undertaken to evaluate various factors related to wet granulation such as the effect of granulating fluid, type of end plate, number of mixing anvils and screw speed. Another objective of these studies was to further understand the level of content uniformity during granulation. The use of water-isopropanol as granulating fluid reduced the sugar solubility in the granulating fluid, creating increased torque during highspeed processing. The granules prepared by using this granulating fluid resulted in the compression of less gritty and smoother tablets compared to water. It was concluded that the water- isopropanol granulating fluid created processing interruption but produced granules with low bulk densities.

Later on, in 1986, Gamlen and Eardley introduced the twin screw extruder in pharmaceutical research, to produce paracetamol extrudates with a combination of excipients i.e., Avicel, lactose and/ or hydroxypropyl methyl cellulose (HPMC) and using water as a granulating fluid [48]. The results revealed that addition of HPMC in the formulation influenced significantly on the extrusion properties. HPMC helped to retain water in its interstitial spaces, reducing frictional forces between extruder and the plate. Micrographs of the extruded formulation with and without HPMC showed similar appearances. Hence, the addition of HPMC improved the extrudability without affecting the extrudate quality. A year later, Lindberg (1987) and later Kleinebudde (1998) also employed twin-screw extruders for the formation of effervescent granules [49,50,51,52,53,54]. Furthermore, TSG has been implemented to perform dry, wet and melt granulation process [55] including scaling-up of continuous granulation coupled with Process Analytical Tools (PAT) by applying Quality by Design (QbD) approaches [56].

Despite the fact the TSG is an emerging technology the majority of the published studies are data-driven and (semi-) mechanistic which investigate the association between the process parameters and the granulation performance using multivariate data analysis. However, the effect of critical processing parameters (CPP) on the growth and kinetics of granule formation is not well established in comparison to batch granulation (e.g., high shear).

**Figure 8:**
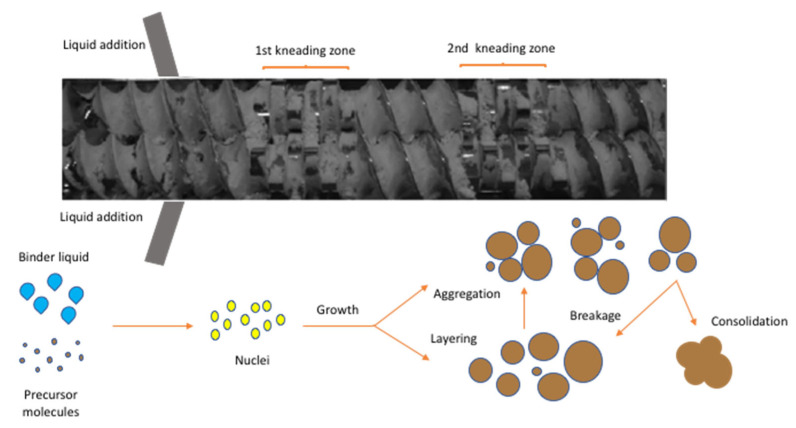
Screw configuration with 12 kneading disks illustrating the formation of granules in TSG (Reproduced with permission from [62], John Wiley and Sons, 2017).

For this reason, theoretical [57,58] and experimental [43,59,60,61] approaches have been introduced to understand the transport, mixing and the fundamental mechanisms in twin-screw wet granulation. As shown in Figure 8, the dry powder is fed in the barrel through the feeding zone while the granulating liquid is added using two nozzles (for each screw). The granule formation takes place through a combination of capillary and viscous forces facilitating particle binding in the wet stage. Subsequently, the wet material is distributed, compacted and elongated in the kneading elements (mixing zone) transforming the particle morphology from small (microstructure) to large (macro structure) irregular and porous granules. Other sub -processes such as aggregation and breakage can also take place during granulation. Eventually, the formed granules leave the discharge zone and are pneumatically fed in a fluidised bed dryer.

A very interesting study was conducted by Dhenge et al. [63] who replaced the top metal barrel of the extruder and replace it with an acrylic glass barrel to visualize the granulation process and identify the possible mechanisms. The authors concluded that there are five regions of granulation behaviour or finished granules classified as under-wetted (dry), nuclei, crumb, granules and over wetted or paste. The under wetted granules were obtained when low amount of granulating liquid was used resulting in un-granulated material. The nuclei refers to the initial wet mass which comprises of small granulating liquid amounts and are loosely bound with weak mechanical properties. The “crumb” denotes again ungranulated or poorly granulated materials which present different strength depending on the type of used elements (conveying of kneading). To the contrary, the “granules” are robust consolidated agglomerates which have a similar behaviour compared to classical granules and can be turned to wetted agglomerates or paste if additional granulating liquid is added. The study included the use of particle image velocimetry and discrete element modelling to prove that the feed rate and viscosity greatly affect the surface velocity of dry materials and wet granules. The actual mechanisms of granulation have been further investigated and elaborated by published work from other researchers [61]. These studies provide information on how the processing parameters can significantly affect the aggregation and breakage mechanisms including the consolidation of the particles.

**Figure 9:**
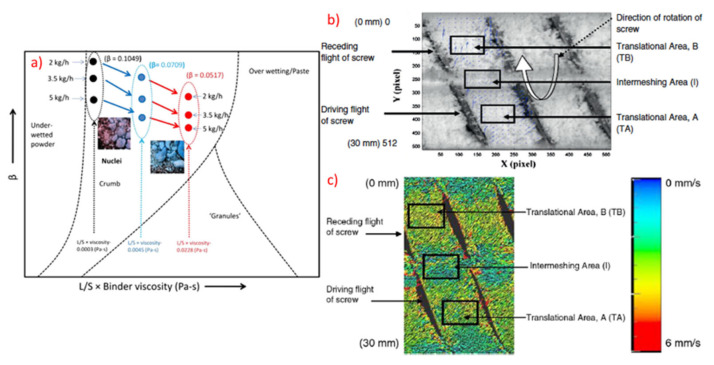
(**a**) Granule regime map for TSG using conveying screws, (**b**) Representation of different areas in the screw channel in 2D and (**c**) Snapshot showing different areas in the screw channel with surface velocity vectors (Reproduced with permission from [63], Elsevier, 2013).

Furthermore, modelling approaches, for example, using novel multi-component population balance model have investigated TSG processing by taking into account the rate processes of aggregation, breakage, liquid addition and consolidation. The linkage of the granulation mechanism with experimental data were used to develop predictive tools [58]. Kumar et al., introduced conceptual model in order to understand and simulate the residence time distribution (RTD) for a particular TSG formulation that could be easily adopted for other excipients [57]. The comparison of the experimental findings and best conceptual model were used to estimate and predict the influence of kneading elements, stagger angle, screw speed and powder feed on RTD (Figure 9).

In 2010, Dhenge et al. conducted an empirical study on a twin – screw extruder TSE with model pharmaceutical formulations focused on the physical properties of the final granules [42]. The authors investigated the effect of screw speed, powder feed rate and liquid to solid (L/S) ratio on the residence time and torque which was found to affect the particle size, the strength, shape and structure of the granules. The most pronounced effect on granule properties was observed with a L/S ratio of 0.4, showing a monomodal distribution of granule sizes with a peak around the 1000 µm mark. At higher L/S, the extra liquid caused an increase in the residence time of material in the granulator, resulting a reduction of undersized and oversized granules. The granules shape and hence the flow was improved due to the increased amount of granulating fluid which helped to achieve stronger liquid bridges between the particles. The powder feed rate influenced the transition and final state of granule properties such as size, shape, structure, porosity, strength, and dissolution time. At a low feed rate the residence time became long, resulted in strong granules with an increased average granule size whereas higher feed rate reduced the granule size. At a powder feed rate of 3.5 and 5 kg/h, the sphericity of the granules was found to be increased. The improved sphericity of granule was related to the increased filling at high powder feed rate which led to increase in shearing forces within the barrel and turned the processed powder to spherical agglomerates. The surface morphology of the granules became smooth by increasing the length of the screw while porosity was decreased. The high feed rate not only increased the granules morphological strength and stability but also affected the dissolution rates. It was concluded that TSG optimisation is a complex process, and the granule properties (size distribution, strength and structure) are affected significantly by the critical processing parameters.

Vercruysse et al. (2012) investigated the operational parameters related to TSG and their effect on the manufacturing process using theophylline as model API [34]. There was no significant relationship of screw configuration and screw speed to the granules morphology, but high number of kneading elements and increased throughput resulted higher torque during granulation. The high torque raised the temperature in the barrel which led to reduced number of fines and less friable granules. The binder was found to be more effective when it was dissolved in the granulating fluid. Increasing the number of kneading elements yielded denser granules with a longer disintegration time and dissolution rate. The findings of this work suggested that the granule and tablet quality can be optimised by adjusting specific process variables. Khorsheed et al. (2017) also investigated the effect of TSG processing parameters on the powder and granule and tablet properties using microcrystalline cellulose (MCC) or mannitol C160 [64]. Their study showed increasing MCC granule size and strength can reduce tabletability and vice versa. Although, mannitol C160 granules did not affect tabletability, particle size reduction had shown significant compactibility improvements. They found a correlation between the yield pressure, plastic and elastic work of the initial powders and changes in tabletability performance as a result of the granulation process. The authors mentioned that increasing the strength and size of granules may cause reduction of the tabletability.

The granule structure is closely related to tablet’s quality attributes and it can be controlled by the selection of appropriate excipients as it was recently reported by Megarry et al. [65]. Allopurinol granulated formulations prepared by wet granulation via both twin-screw and high shear processing. By using different mannitol grades (200SD and 160 °C) a polymorphic transition during processing, containing mostly β-mannitol, was found. The 200SD granules had a needle-shaped morphology with high porosity and specific surface area, which led to poorer flow properties but higher tablet tensile strength. Furthermore, 200SD granules presented lower d10 values compared to 160C while the L/S ratio did not affect the size as it was observed for the 160 °C. The study suggested that the understanding of specific excipient grade and their effect during processing is crucial to optimise tablet manufacturability.

Lute et al. (2018) investigated the influence of varying barrel fill levels on the mean residence time, granule properties and tensile strength of tables using MCC and lactose [66]. They reported that specific feed load or powder feed volume directly affects the granule size and shape. Increasing fill levels of MCC inside the extruder barrel caused shorter residence times along with decreased granules size. On the other hand, lactose maintained its granule size at all fill levels. Again, the usage of a specific pharma excipient and their processing parameters are crucial parts of the granulation optimisation process.

Twin screw dry granulation is considered more effective for granule manufacturing as it limits heat exposure to only one-barrel zone, much shorter than melt granulation. Lui et al. (2017) studied formulations containing different polymeric binders (AF15, Kollidon VA 64^®^, Soluplus^®^, Kollidon SR^®^), with glass transition temperature less than 130 °C [67]. Granulation of the primary powders with some degree of moisture was found to be beneficial for processed polymers with high glass transition temperatures. Selected polymer particles are more prone to soften and flow under frictional forces if their T_g_ was closer to the barrel zone temperature in the kneading section. Because of this, the Barrel temperature is often set to match the T_g_ of the processing polymer to allow for successful granulation. Screw speed was a major cause for friction heating while the kneading block offset was only minor in its influence on the granulation process. According to their results, a higher screw speed tended to increase the particles size, producing bigger chunks (>3350 µm). Conversely, an increase of moisture content in the excipient resulted in smaller particle size distribution. So, successful granulation can be achieved by varying the processing parameters and pre-formulation studies will allow one to quickly optimise the process.

Ye et al. (2019) used twin screw dry granulation to improve the flow property of moisture sensitive materials [68]. They produced dry granule formulations using four different APIs processed with Klucel^TM^, Ethocel^TM^, and magnesium stearate for sustained release. A Design of Experiment (DoE) was employed to determine the effect of different processing parameters i.e., screw speed, feeding rate, barrel temperature and screw configuration on the product properties (flow properties, particle size distribution and dissolution time). It was revealed that an increased screw speed related to a higher percentage of medium size granules while a negative correlation was found between the amount of large size granules and screw speed. This was attributed to the decrease of the mean residence time due to the high screw speeds and the subsequent reduction of the kneading effect on primary powders and the formation of smaller granules. Higher feeding rates improved the flow properties of the powders and decreased angles of repose were obtained. The morphology of granules affected the powder flow where long stripe shape exhibited poor flowability compared to round shape particles. Finally, drug release was affected by the binder content in the granule and presented significant variations i.e., large stronger granules with more binder showed relatively slow release. On the other hand, small amount soft binder provided less particle adhesion which resulted in faster disintegration and hence faster dissolution rates. The continuous processing, simplicity of operation, absence of milling, suggest that twin-screw dry granulation TSDG is more effective compared to other conventional dry granulation approaches.

Kallakunta et al. (2019) applied heat assisted dry granulation using a twin screw extruder to formulate sustained release granules [69]. Granulation feasibility was studied with different binders (e.g., Klucel™ EF, Kollidon^®^ VA64), sustained release agents (e.g., Klucel™ MF, Eudragit^®^ RSPO) and diluents at various drug loads. The processing conditions were below the melting point or glass transition temperature of the formulation ingredients. They found a size correlation to the binding capacity of the excipient. Formulations with Klucel prepared granules with a size of around 250 µm whereas Kollidon^®^ VA64 promoted the formation of finer granules related to its own particle size (20 µm). Hence, the good binding properties of the ingredients in the formulation, facilitated easier granule formation and larger granule size. On the other hand, formulations consisting of only Klucel™ MF showed minimal erosion (approximately 5%) over 24 h. The drug release was found to be incomplete in these formulations, due to the high viscosity of Klucel™ MF. However, the excellent binding properties and viscosity of Klucel™ MF resulted in dosage forms with good matrix integrity, which then became one of the controlling factors for drug release. Conversely, formulations with Kollidon^®^ VA64 reported to undergo greater erosion which destabilised the tablet matrix and led fast drug release. In summary, heat-assisted dry granulation could be applied for continuous twin-screw granulation, which may ameliorate the process constraints and stability problems in conventional granulation techniques. However, the careful polymer selection is crucial to achieving the desired physiochemical properties for the end product.

Unlike wet and dry granulation, melt granulation offers several advantages for processing pharmaceutical actives. Twin-screw melt granulation is carried out at higher temperatures than traditional batch melt granulation and thermoplastic polymers can be used as binders. This has a clear benefit, considering the limited number of traditional binders that are suitable for use in conventional granulation processes. The process of twin-screw melt granulation is also advantageous as it eliminates the need for organic solvents and water, while it is cost effective and environmentally friendly. As a totally water-free process, twin-screw melt granulation is suitable for drugs that undergo hydrolysis or degradation in the presence of water but may not be suitable for some heat-labile API.

Ven Melkebeke et al. (2006) studied twin screw melt granulation for the manufacturing of immediate release formulation using two grades of polyethylene glycol (PEG 400 and 4000) as a binder [20]. The authors described the importance of granulation temperature on its dissolution properties. A high yield and fast dissolution rate were obtained only at a processing temperature near the melting point of PEG. Post granulation characterisations showed that a homogeneous dispersion of the BCS class II drug within the polymeric matrix created a micro-environment around the drug particles enhancing the dissolution rate. The addition of small surfactant amounts (polysorbate 80 or Cremophor) helped to achieve a complete drug release within 10 minutes. A high drug content required greater PEG and surfactant content to obtain 100% drug release.

Batra et al. (2017) investigated polymeric binders with high melting points (180 °C) for improving tabletability of APIs using twin screw melt granulation [70]. For the purposes of the study metformin hydrochloride and acetaminophen were used as active ingredients and several polymers i.e., hydroxypropyl cellulose, hydroxypropyl methyl- cellulose, polyvinylpyrrolidone and methacrylate-based polymers, including Klucel^®^ EXF, Eudragit^®^ EPO, and Soluplus^®^ as binders. The prepared granules demonstrated good tensile strength during tableting even with polymer concentrations as low as 10% *w/w*. As the melting temperature of acetaminophen is below 180 °C, its TSG temperature was achieved at 130 °C, and even at that temperature, extruded granules provided acceptable compatibility of >2 MPa, suggesting that compressed tablets could withstand manufacturing and end-use stresses during coating, packaging, transportation, and handling. The work demonstrated that a pool of polymeric binders can be successfully use for twin-screw melt granulation and further exploited in the future.

**Figure 10:**
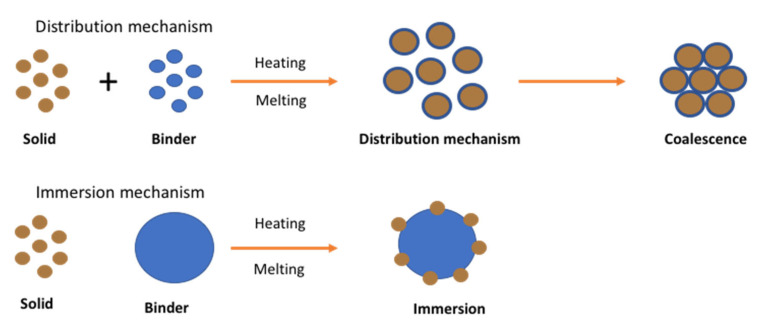
Nucleation mechanism of melt granulation (Reproduced with permission from [71], Elsevier, 1996).

Unlike wet or dry granulation, in melt granulation the growth mechanism involves an additional nucleation mechanism known as immersion or distribution as shown in Figure 10.

The mechanism was studied by Monteyne et al. (2016) for the development of immiscible drug-binder formulations [72]. They implemented thermal analysis, rheological characterisation and microscopic images to reach an in-depth understanding of material behaviour during agglomeration. They reported that the distribution of the binder in the immiscible blends caused a double T_g_ and a clear loss peak in the thermogram. The binder was found to act as a separate phase favouring efficient binder distribution where a thin binder layer with restricted mobility is formed on the surface of the primary drug particles during granulation. Then it is covered by a second layer with improved mobility when the binder concentration is sufficiently high. The granules manufactured with 20% (*w/w*) Soluplus^®^ or higher became smaller and more spherical as a function of temperature whereas a lower binder concentration resulted in larger and more needle-shaped granules. The study showed strong evidence of the binder distribution during TSG which strongly affected the granule characteristics.

In another study, the same group carried out TSG studies with Soluplus (SOL) and metoprolol (MTP) or caffeine [73]. In this case, thermal analysis showed only one T_g_, indicating a highly miscible system. A high barrel temperature was used to obtain granules larger than 500 µm which also showed minimum torque fluctuation during granulation. Surprisingly, granules prepared with 20% or more binder concentration resulted large aggregates at processing temperatures <90 °C. Prepared granules with caffeine-Soluplus blends displayed a broad particle size distribution over the different binder concentrations and was not affected by the process temperatures whereas the granule size distribution of the MPT/SOLblends appeared narrower and shifted towards lower particle sizes with an elevated granulation temperature.

## 5. Twin Screw Granulation and Continuous Manufacturing

The traditional manufacturing of medicinal products in the pharmaceutical industry uses batch processing, where every single unit operation is conducted separately. The adoption of continuous manufacturing (CM) from pharmaceutical industry is relatively new and it takes place in a stepwise manner. On the other hand food, petrochemical, polymer and oil refining industries have been undertaking CM operations for decades and produce large volume production at a cost effective manner [12,74,75,76]. The term “continuous” means a required process may run for an extended period, with raw material constantly fed into the process. The pharmaceutical industry has faced many obstacles in attempting such a continuous production method on a day-to-day basis. The main perceived issue is the industry’s rigid structure, because of the strict supervision of regulatory agencies such as the U.S. Food and Drug Administration FDA in the U.S. or the European Medicines Agency (EMA) in Europe [77,78,79].

Despite the fact that there are no regulatory hurdles for implementing continuous processes pharmaceutical industry needs to reform regulatory framework, pharmaceutical equipment design and operation. Some pharmaceutical companies plan to convert 70% of their production lines to continuous manufacturing to allow a more efficient product and process development as part of the product operation. CM processes are usually operating at or near steady state which allows a clsosed-loop control of the entire process and thus to a robust and reliable operation. They usually reach steady state in a few minutes enabling the application of a true QbD manufacturing approach.

**Figure 11:**
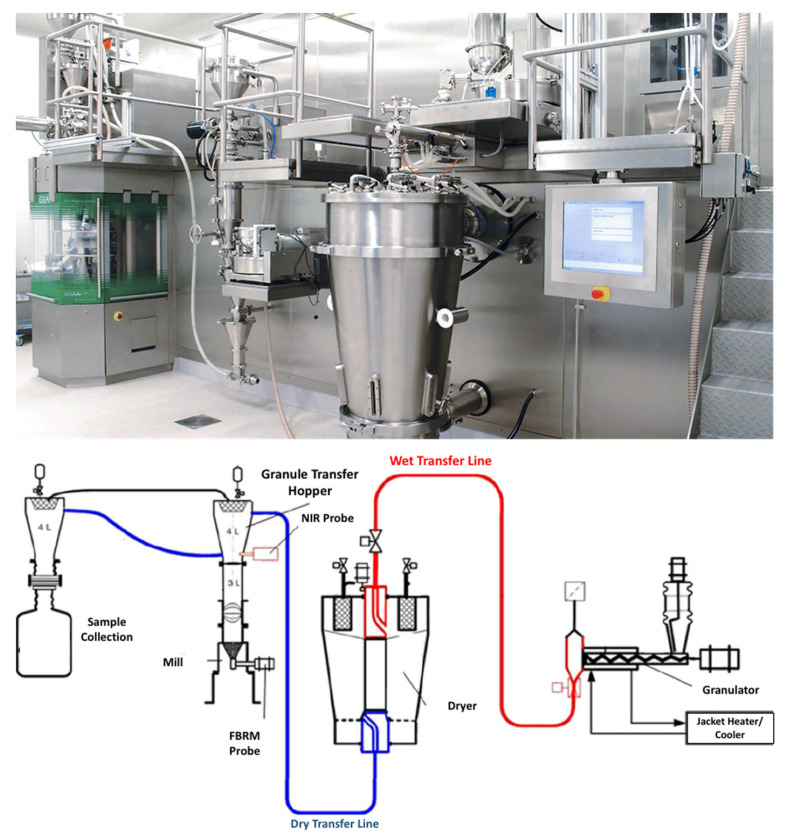
ConsiGma^TM^ continuous high shear granulation process by GEA Group (images provided by the manufacturer).

Nevertheless, TSG is an ideal example of such a process that has successfully been introduced for CM production. Recently, GEA introduced a continuous platform for oral solid dosage forms which integrates multiple operation units comprising of granulation, drying and tablet compression. As shown in Figure 11, the process involves the dosing and mixing of raw materials suing multiple feeders, followed by the high shear granulator for mixing, wetting and granulation through the coupling with a highly accurate dosing system (e.g., water, solvents, binder solutions). Subsequently the granules are continuously transferred (e.g., vacuum or gravity) to a fluidized bed dryer for drying and milled to achieve the desired particle size and content uniformity. The drying process is monitored with an on-line moisture LightHouse^TM^ probe. Prior to tablet compression in a rotary tablet press the dried powders are blended with the external phase (e.g., lubricants, disintegrants, fillers). The tablet press comprises of 6 compression modes such as compression to equal porosity, Exchangeable Compression Module with a wash-off-line capability which are coupled with advanced in-line PAT sensors and a control system. The technology is versatile and processed amounts vary from 500 g for R&D purposes up to 100 Kg/h.

**Figure 12:**
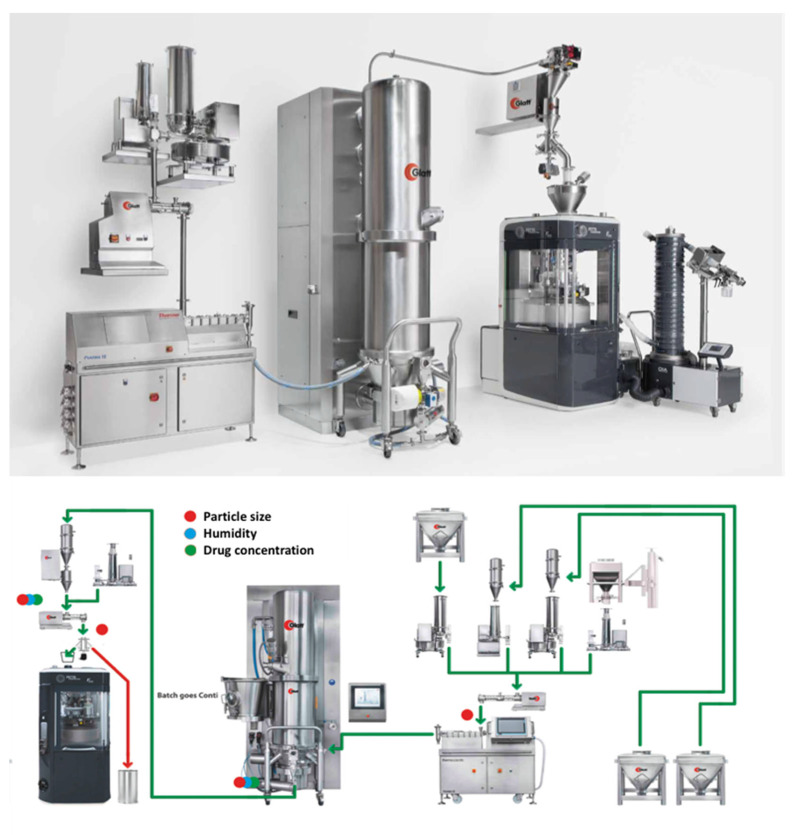
MODCOS continuous high shear granulation process by Glatt (images provided by the manufacturer).

A similar processing technology known as Modular Continuous System MODCOS has been developed by Glatt for the conversion of the batch mode to a continuous fluid bed system. As shown in Figure 12, loss in weight feeders is used for the dosing of the active drug substance and excipients through vacuum conveyor in a twin-screw extruder to produce medium to high density granules. The wet granules are pneumatically conveyed into the process insert of the fluid bed via a specially designed transfer line, where they are continuously dried to the required moisture level. Before the granules are compressed in tablets with other ingredients a dry mixer is used to achieve homogeneous mixing. A key advantage of the MODCOS line is the narrow retention time distribution during drying while a sophisticated discharge system facilitates complete emptying of the process chambers and thus preventing any cross-contamination with consistent product quality. The continuous line is coupled with a range of PAT tools including two Near–Infrared (NIR) probes for determine content uniformity at the end of the extruder or the discharge system. A moisture probe is used at the fluid bed dryer discharge point and a particle sizer probe is inserted at the powder discharge points (feeders).

A unique feature of the continuous line is the intelligent control system where all the process parameters are controlled together and continuously monitored to provide the basis for automatic process control. The manufacturing process is controlled by recipes which include the distribution of residence times while the data for all process unit can be displayed as tables of graphs.

In batch manufacturing of solid dosage form there is a limited process understanding and control which requires the application of more intensive and advanced manufacturing processes. Continuous granulation processing is fully automated and thus scale up issues related to batch manufacturing are no longer encountered. Often, in batch granulation, the active substances tend to agglomerate especially when they present highly cohesive properties. This problem can be easily mitigated in continuous granulation by combining high shear co-milling of the drug substances and excipients followed by low -shear blending. The powder blends are immediately compressed to produce tablets and, unlike in batch processing, do not allow time for particles to re-agglomerate. CM lines can be built in a flexible way for the processing of multiple formulations (products) through careful consideration and optimal design. Continuous granulation lines can be easily adopted for both dry and wet processing which eventually results a good return on the initial investment. Other advantages of continuous granulation processing include the following [80]:

- Enhanced development approach by implementing QbD approaches and incorporating PAT tools
- Reduce risk of manufacturing failure and prevent drug shortages
- Decreased risk of out of specification failures both for intermediates and finished products
- Flexibility by using the same system to develop a manufacturing process
- Effect on supply chain by increasing supply speed and react to market demands
- Agility and reduced scale-up efforts
- Real time quality assurance-secure quality attributes and measure critical quality parameters
- Reduction of capital and operational costs, environmentally friendly
- Cost reductions in R&D, product transfer and productivity
- Reduced system’s footprint

Continuous TSG is an attractive manufacturing process for the development and commercialization of marketed products. Currently there several commercial products on the market (small molecules) as shown in Table 1.

## 6. QbD Approaches in Twin-Screw Granulation

The benefits of using QbD approaches in pharmaceutical manufacturing has been recognized and promoted by regulatory bodies such as the Food and Drug Administration (FDA) and International Conference on Harmonisation (ICH), combined with PAT principles and closed loop quality assurance [83,84,85,86]. CM lines have been implemented in the pharmaceutical industry for the manufacturing of the drug product Orkambi by Vertex for the treatment of cystic fibrosis in 2015. There are only a few studies in literature related to QbD approaches coupled with PAT tools related to twin-screw granulation.

The first report was conducted in 2014 by Fonteyene et al., who investigated the effects of variation in raw material properties on the Critical Quality Attributes (CQAs) of granules produced by wet granulation followed by tableting [87]. By using a powder-to-tablet wet granulation line a model formulation of theophylline–lactose–PVP (30–67.5–2.5%) was investigated while the process parameters were kept steady. For the purposes of the study, seven grades of theophylline were processed, and the features of granules/tablets were evaluated. The granule particle size for all experiments showed a bimodal distribution with granules being in–spec containing either large amounts of fines (>150 μm) or large amounts of oversized particles (>1400 μm). The differences were directly correlated to the initial particle size of the theophylline grade. The granules obtained with fine powders presented higher bulk density and lower tapped density compared to the initial powder blend suggesting that no tapped volume reduction can be produced when granule powders have high tapped densities.

**Figure 13:**
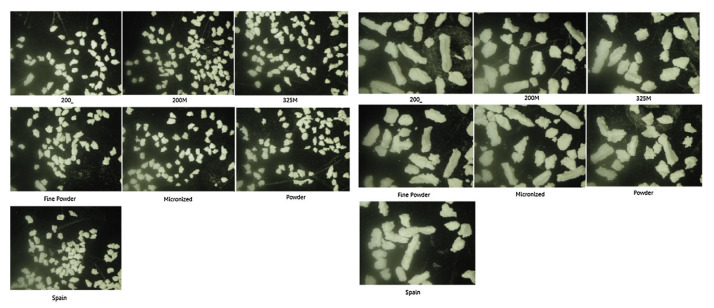
Pictures of two sieve fractions of the seven granule loads (500–710 μm and 1000–1400 μm) (Reproduced with permission from [87], Elsevier, 2014).

As shown in Figure 13, the granule morphology was needle-shaped while for the small sieve fractions more spherical particles were observed without however been able to identify any differences for the granules of the various theophylline grades.

Principal Component Analysis (PCA) applied for the content uniformity showed that smaller granules present more lactose monohydrate while larger granules more theophylline. The investigations on the processability showed that theophylline powders play a key role in feeding with large powders pushing the injectors out of the granulator barrel. Regarding the tableting process a direct relation between the granule size and compression forces was observed with small size fractions (<150 μm) requiring higher compaction forces.

Maniruzzaman et al. applied a QbD approach by introducing a DoE to investigate the effect of formulation composition in a wet extrusion granulation process [88]. TSG was conducted using blends of polymer/inorganic excipients (hydroxypropyl methylcellulose and magnesium aluminometasilicate-MAS) as carriers and PEG as the binder to produce Ibuprofen (IBU) granules. The MAS/polymer ratio, PEG amount (binder) and L/S ratios were set as the independent variable while the dissolution rates, mean particle size (D_50_) and the loss on drying (LoD) of the extruded granules as the dependent variables. The morphology of the obtained granules appeared spherical for all processed batches compared to the needle -shaped IBU with the D_50_ particle size varying from 100–300 μm. Dynamic vapour sorption analysis showed a reversible water uptake of all batches and provided evidence that the inorganic excipient prevented significant water uptake. The content uniformity was assessed using Confocal Raman mapping and demonstrated excellent IBU distribution within the granules which was partially amorphous as a result of the TSG processing. The DoE analysis revealed that the PEG amounts and the L/S ratio had a significant effect on both the IBU release and the LoD. The granule particle size distribution was found to be affected significantly by the MAS/polymer ratios but also the binder amounts.

**Figure 14:**
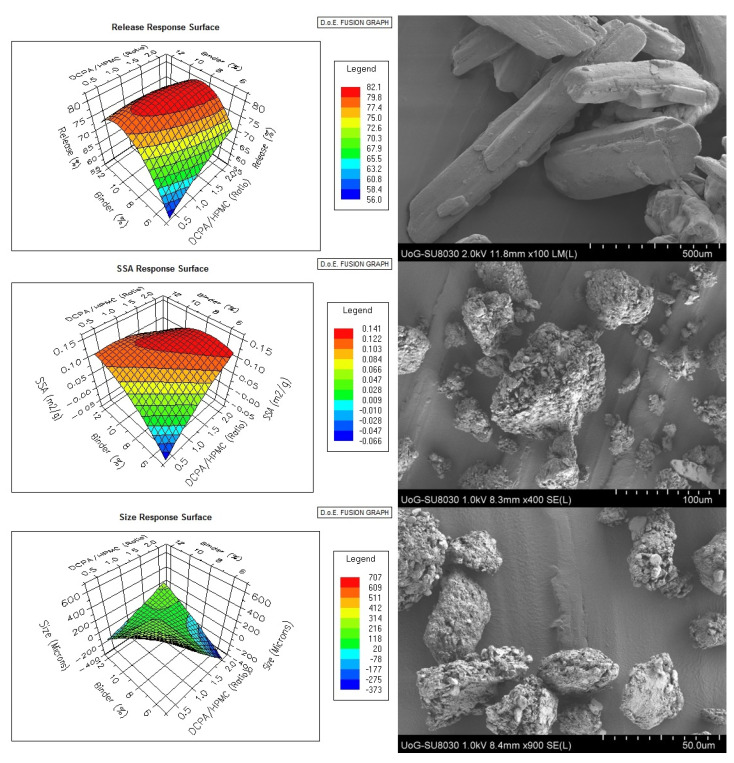
Response surface plots of Ibuprofen (IBU) release, specific surface area and particle size distribution dependent variables (**Left**). Scanning electron microscopy images of (**A**) bulk IBU, (**B**) F2 granules (DCPA/Polymer 1.0, Binder 8.0%, L/S ratio 0.30) and (**C**) F10 granules (DCPA/Polymer 1.0, Binder 8.0%, L/S ratio 0.30) (**Right**) Reproduced with permission from [89], Elsevier, 2017.

The same group conducted an identical study using DoE to investigate the effect of the excipient composition, binder amount and L/S ratio (independent variables) on drug dissolution rates, median particle size diameter and specific surface area (dependent variables), as shown in Figure 14 [89]. This time, ethanol was used as granulating liquid instead of water.

The use of a different liquid resulted in the formation of larger granules with D_50_ varying from 200–583 μm. For most of the batches, a monomodal particle size distribution was obtained while the granule morphology appeared spherical as before. This time all independent variables were found to have a significant effect on the drug dissolution rates and particle size distribution while the two-way interactions were identified after the data integration suggested a complex granulation process.

The granule specific surface area was only affected by the MAS/polymer ratios and the PEG amounts at a significant level. Interestingly, the water insoluble IBU demonstrated relatively fast release rates within 2 h (pH 1.2) due to the increased amorphous content which resulted due to the solubilization effect of ethanol and the drug absorption within the porous network of the inorganic MAS excipient.

Another comprehensive study was conducted by Grymonpré et al., who investigated the impact of critical process parameters CPP) and critical material parameters (CMP) on the CQAs in twin-screw melt granulation process followed by milling and tableting of the formed granules [90]. Two active substances, Acetaminophen (APAP) and hydrochlorothiazide (HCT) were co-processed with a range of hydrophilic polymers such as Kollidon VA64, Soluplus (SOL), Eudragit EPO and Affinisol grades (15 LV, 4M). The processing temperatures were affected by the glass transition of the binders and, for the HPMC grades higher temperatures were used due to their complex viscosities.

**Figure 15:**
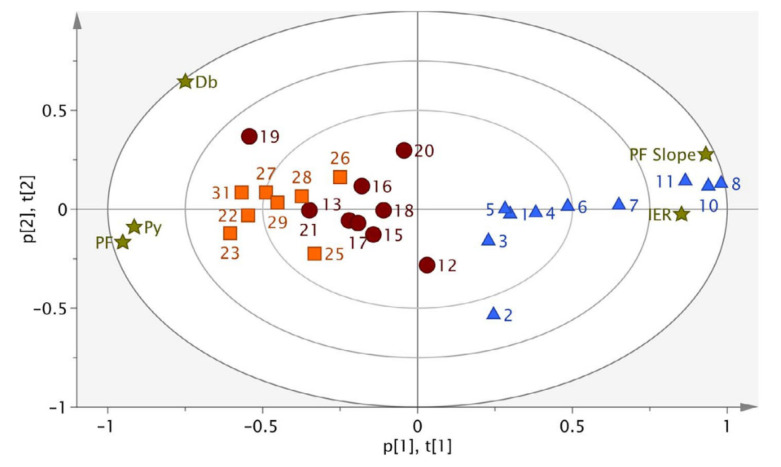
PC1 vs. PC2 biplot of the determined compaction properties (loadings) for the experimental runs of the DOE (scores). The numbers represent corresponding experimental run of HCT-EPO formulations (blue triangles), HCTSOL formulations (dark red circles) and HCT-VA64 formulations (orange boxes), plotted against loadings (star shaped) for which PF represents the plasticity factor, IER the anti-correlated in-die elastic recovery, PF slope the slope of the plasticity factor over 4 compaction pressures, Py the heckel value and Db the fragmentation factor. Reproduced with permission from [90], Elsevier, 2018.

The milled granules presented lower moisture content (<1%) with a consistent shape and good flowability. More fines were received when EPO and Affinisol were used as binders, but overall, all polymers were suitable for melt granulation processing. SOL and VA64 outperform the rest of the binders due to their high milling efficiency and resistance to forming fines. Compatibility studies showed identical performance for both drugs irrespective of the polymeric binder. The tabletability studies (Figure 15) showed significant improvement of the melt granulated products compared to physical mixtures. The T_g_ of the polymer was found to affect the extrusion processing especially for VA64 which resulted in increased torque, while binders with low T_g_ (e.g., SOL, EPO) facilitated a smoother granulation process. Similarly, the tableting process was also affected by the binder grade where EPO showed less fragmentation and higher elastic deformation during tablet compaction in comparison to SOL and VA64. Overall, the study demonstrated that twin-screw melt granulation was robust for both low and high drug loading processes and CQAs were well identified.

In another study, the same group applied a QbD methodology using the continuous ConsiGma^®^ extrusion line to investigate the formulation optimization of twin-screw granulated composition of various binary filler/binder grades and ratios [91]. By using multiple linear regression models, the authors were able to understand the impact of the filler/binder properties on the granule and tablet CQAs. A DoE with 27 batches was combined with PCA plot to reduce large date sets and identify similarities or differences of materials with different chemical characteristics. The overall scope of the study was to identify the impact of materials on CQAs and consequently develop predictive formulation models with suitable characteristics for the processing of APIs with unfavourable properties. For example, the study demonstrated that the granule particle size was not affected by the original particle size or the water uptake of the filler. The granule flowability was found to be influenced by the binder at higher concentrations but not affected by the filler properties. The granule friability decreased when PVP was used at higher concentrations. Finally, the tabletability was not affected by the grade of the filler which showed no impact at all. To the contrary the binder grade and the use of higher PVP concentrations demonstrated a significant effect on the improved tabletability. When compared to other binder grades it was found that the bulk density and specific surface area also plays a role on the deformation mechanisms, and hence tabletability.

## 7. Process Analytical Technology (PAT)

PAT are defined as “tools and systems that utilize, analyze and control real-time measurements of raw and processed materials during manufacturing, to ensure optimal processing condition are used to produce final product that consistently conforms to established quality and performance standards” [85]. The goal of PAT is to enhance understanding of and control the manufacturing in-process, making it consistent with our current drug quality system. As such, quality cannot be tested into products; it should either be built-in or by design.

Design space is defined by the CPPs identified from process characterization studies and their acceptable ranges. These parameters are the primary focus of on-, in-, or at-line PAT applications [92]. In most cases, spectroscopic techniques, such as Raman spectroscopy, UV-Vis spectroscopy and NMR (nuclear magnetic resonance) are commonly used. Besides the foregoing, near-infrared spectroscopy (NIR) [93], Nano metric temperature measurement (MTM) [94] and tunable diode laser absorption spectroscopy (TDLAS) are widely applied tools in the pharmaceutical manufacturing field and play important roles in real-time monitoring of the processes used. Among these, NIR has drawn great attention in the industry because it is a rapid, non-invasive analytical technique and there is no need for extensive sample preparation [95,96,97,98]. Typically, it is used for the identification and characterization of raw materials and intermediates, analysis of dosage forms manufacturing and prediction of one or more variables in the process line or the final product stream(s) on the basis of on-line, in-line or at-line spectroscopic measurement [99]. The increasing interest in QbD combined with tools DoE, PAT and risk assessment approaches, has transformed pharmaceutical processes from and been adopted by pharmaceutical industry in order to address quality, manufacturing issues.

The framework of PAT is intended to support the innovation and efficiency in pharmaceutical processes manufacturing and quality assurance [100]. This includes systems that can be employed to design, analyse and control manufacturing by measuring critical quality and performance attributes in a timely manner. This involves measurements of raw and in-process materials aiming to ensure the quality of the finished products. PAT is not only a way to implement real-time release testing but also to effectively detect failures and help understanding the manufacturing processes. Due to the versatility of PAT tools, relevant information can be obtained by monitoring a range of physical, chemical and biological attributes. This can be achieved mainly by four key components which include:

- Multivariate tools for design, data acquisition and analysis which help to build scientific understanding but also to identify CPPs and critical material attributes (CMAs) which eventually leads to process understanding while the generated information is integrated into the process control.
- Process analytical chemistry tools can effectively provide real-time and in situ data for the processed materials and process. The process measurements can also be near real time (e.g., on-, in and at-line) in invasive or non-invasive manner.
- Process monitoring and control involves the design of process controls and development of mathematical relations that provide adjustments to achieve control of all critical attributes.

Continuous process optimization and knowledge management relates to data collection and analysis from generated databases over the life cycle of the finished product.

### Process Analytical Technology (PAT) in Extrusion Granulation

PAT tools have been successfully implemented in continuous wet granulation production lines, namely ConsiGma^®^ developed by GEA Pharma [101]. The process consists of 5 locations where the critical quality attributes are measured. The first and the third measure the blend uniformity of the processed powders and the moisture content of the dried granules while the second monitors the moisture distribution of the formed granules. 

A NIR sensor is coupled to the 1st location to allow accurate control of the powder feeding and blending and if possible to provide feedback for controlling blending operations [102,103,104,105,106]. Similarly, NIR probes were used to measure the moisture content and distribution.

**Figure 16:**
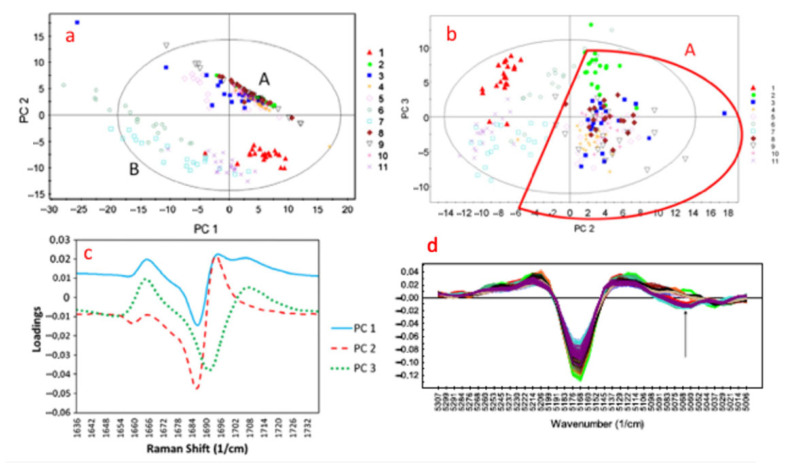
(**a**) Raman spectra: PC 1 (47.12%) versus PC 2 (26.95%) scores plot, (**b**) Raman spectra: PC 2 (26.95%) versus PC 3 (16.24%) scores plot, (**c**) Raman spectroscopy: PC A loadings plots of PC 1, PC 2, and PC 3, (**d**) NIR spectroscopy: Second derivative of NIR spectra. Coloured applied as above for Cluster A (dashed line) and Cluster B (full line) (Reproduced with permission from [101], Elsevier, 2012.

Fonteyne et al. introduced a combination of PAT tools using Raman, NIR and photometric imaging technique for the evaluation of the powder-to-tablet ConsiGma^®^ production line [102]. The aim of the study was to acquire solid-state information and granule size distribution data and in turn use them to predict a range of granule properties such as moisture content, bulk/tapped density and flowability. As shown in Figure 16 the Raman and NIR spectra were collected to apply PCA and determine whether the formed granules contained theophylline monohydrate. Similarly, a PC plot was used to analyse all data collected through 11 DoE experiments when using the FlashSizer 3D process for the estimation of granule size and particle distribution.

The same group conducted a separate study in order to obtain an in-depth understanding of the twin-screw extrusion granulation and fluidise-bed drying processes using Batch Statistical Process Monitoring (BSPM) principles [106]. For the purposes of the study, the group implemented multivariate data analysis in terms of PCA, Partial Least Squares regression (PLS) and their various extensions such as multiblock PCA/PLS and orthogonal PLS. The work demonstrated how multivariate data analysis can be routinely used to generate data from the variable, monitored by the univariate sensors for a continuous granulation process and how the BSMP concepts are used to monitor variables in order to identify operational variations.

Madarász et al. studied real-time feedback control of twin-screw wet granulation using dynamic image analysis [107]. In a typical granulation process of lactose and starch blends a process camera was coupled with image analysis to monitor the particle size distribution of the obtained granules. The real-time feedback control was implemented by controlling the feeding rate of the granulating liquid (fed into the TSG through a peristaltic pump) through a PC.

**Figure 17:**
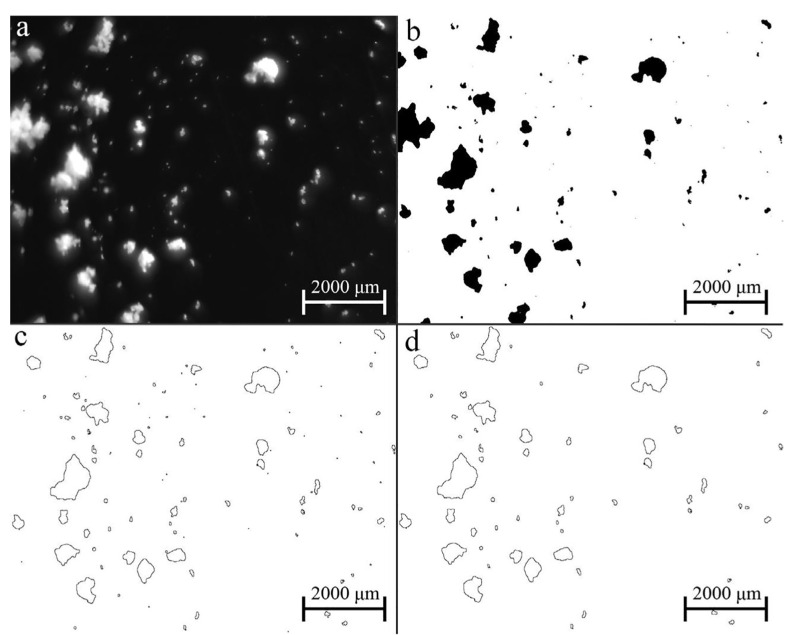
Stages of image processing: (**a**) Raw image; (**b**) Pre-processing; (**c**,**d**) Post-processing (Reproduced with permission from [108], Elsevier, 2018.

As shown in Figure 17 the image analysis software consisted of three main stages:

- Pre-processing: Greyscale filter, Binarization.
- Post-processing: Excluding particles on the edges of the image, Edge detection, Removing noise.
- Analysing and classification: Particle count, Determining particle characteristics (minimum and maximum calliper diameter, aspect ratio), Classification, Summarization.

**Figure 18:**
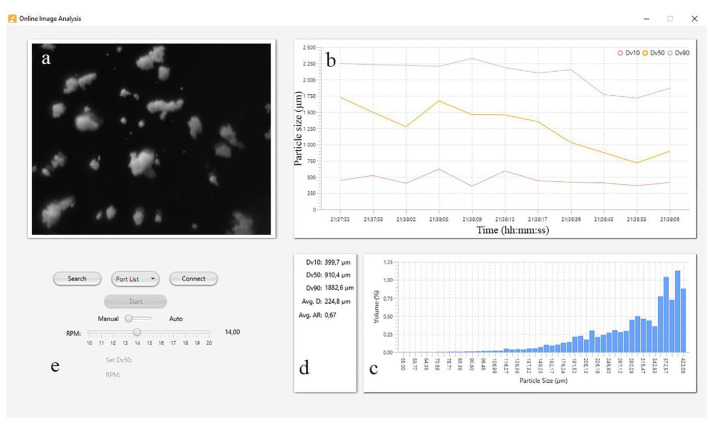
User interface of the developed Online Image Analysis software. (**a**) Current picture being analysed (**b**) Dv10, Dv50 and Dv90 over time (**c**) Particle size distribution (**d**) Current Particle size and Average Diameter (**e**) Control Panel for the peristaltic pump.

The peristaltic pump is controlled through the image analysis software by a manual RPM or an auto mode (Figure 18) where the software controls the pump’s rotation speed via a P controller. By setting the desired granule particle size (e.g., D_50_: 1200 μm) the granulation process was tested by simulating different events including system start up and pump malfunction. Eventually the system could automatically adjust the granule particle size at the predefined value.

In-line monitoring via image analysis was carried out by Sayun et al., who used a TSG with two different screw configurations and various L/S ratios [107]. The real-time high speed imaging system featured a red-green-blue light that targeted the sample creating 3D images with a capability to record particles size distributions ranging from 50–3000 μm. The work revealed that the increase in the fraction of fines corresponded to the L/S ratio in both screw configurations.

**Figure 19:**
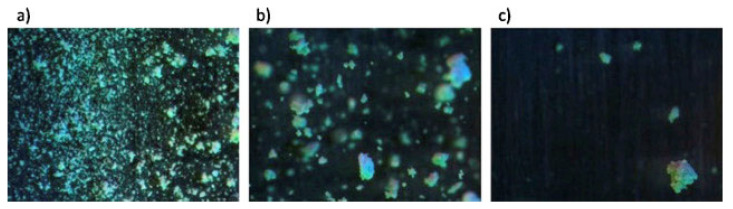
Representative images captured during experiments using 7KE90 configuration at L/S ratio of 0.15 (**a**), 0.25 (**b**), 0.30 (**c**) (Reproduced with permission from [109], Elsevier, 2018).

It was also found that the screw configuration imparts a strong effect on granule porosity while increases in L/S ratio results in decreasing porosity. The authors observed that the small window of imaging (Figure 19) for capturing granule particles resulted in measurement fluctuations originated from powder and liquid feeding methods. The recorded d10 values presented less variations compared to d50 and d90 but were prone to L/S variations.

Rehrl et al. introduced the concept of using a soft PAT sensor in order to control three different continuous processing lines such as HME, direct compression and wet granulation [109]. By measuring the concentration of the API at specific locations using NIR probes (for example, directly after granulation) it was able to predict the concertation of the drug in the feeder. The concentration prediction from on-line spectral measurements (at specific regions) could be done by constructing calibration curves at various *w/w* % and combined PLS regression models. The developed PLS model had a R^2^ of 98.3% for the validation experiments carried out at flow rates of 10–20 Kg/h. These experiments revealed the dependence of the wet granulation process on the feeder excitation.

## 8. Conclusions

Even though twin-screw granulation is a relatively new process in the pharmaceutical industry, it represents an excellent paradigm of pharmaceutical processing that combines principles of QbD and PAT monitoring for process and quality control, while allowing for continuous manufacturing. The flexibility in terms of screw configuration, temperature profiles, feeding site of granulating liquid, produced yield and downstream processing renders TSG advantageous in comparison to conventional granulation processes. However, there is still a lack of adequate association between the experimental findings and theoretical prediction regarding material transport and kinetics in twin-screw granulation. Nevertheless, TSG is one of the few pharmaceutical processes that has proved its potential and applicability for the commercialization of finished products through the implementation of continuous manufacturing. The existing marketed products pave the way for the further exploitation of TSG and the development of novel drug formulations.

## Figures and Tables

**Table 1:** Marketed products using continuous TSG processing [81,82].

Name	Company	Disease	Year
Orkambi	Vertex	Cystic fibrosis	2015
Symdeko	Vertex	Cystic fibrosis	2018
Prezista	Janssen	Anti-HIV	2016
Verzenio	Eli Lilly	Advanced breast cancer	2017
Daurismo	Pfizer	Acute myeloid leukaemia	2018
Lorbrena	Prizer	Metastatic lung cancer	2018
Tramacet	Johnson & Johnson	Additive disorder	2017
